# Landiolol for Treatment of New-Onset Atrial Fibrillation in Critical Care: A Systematic Review

**DOI:** 10.3390/jcm13102951

**Published:** 2024-05-17

**Authors:** Bruno Levy, Michel Slama, Ines Lakbar, Julien Maizel, Hiromi Kato, Marc Leone, Motoi Okada

**Affiliations:** 1 Service de Médecine Intensive et Réanimation Brabois, CHRU Nancy, Pôle Cardio-Médico-Chirurgical, Université de Lorraine, 54511 Vandœuvre-lès-Nancy, France; 2 Intensive Care Unit, Amiens Picardie University Hospital, 80054 Amiens, France; slama.michel@chu-amiens.fr (M.S.); maizel.julien@chu-amiens.fr (J.M.); 3 Department of Anesthesiology and Intensive Care Unit, Hôpital Nord, Assistance Publique Hôpitaux de Marseille, Aix Marseille University, 13385 Marseille, France; ines.lakbar@chu-montpellier.fr (I.L.); marc.leone@ap-hm.fr (M.L.); 4 Department of Anesthesiology and Intensive Care, Hôpital Bicêtre, Assistance Publique Hôpitaux de Paris, 78 rue du Général Leclerc, 94270 Le Kremlin-Bicêtre, France; mynameishiromikato@hotmail.com; 5 Department of Emergency Medicine, Asahikawa Medical University, Asahikawa 078-8510, Japan; motoy@asahikawa-med.ac.jp

**Keywords:** new-onset atrial fibrillation, supraventricular tachycardia, postoperative atrial fibrillation, beta-blockers, landiolol, critical care setting

## Abstract

**Background**: new-onset atrial fibrillation remains a common complication in critical care settings, often necessitating treatment when the correction of triggers is insufficient to restore hemodynamics. The treatment strategy includes electric cardioversion in cases of hemodynamic instability and either rhythm control or rate control in the absence of instability. Landiolol, an ultrashort beta-blocker, effectively controls heart rate with the potential to regulate rhythm. Objectives This review aims to compare the efficacy of landiolol in controlling heart rate and converting to sinus rhythm in the critical care setting. **Methods**: We conducted a comprehensive review of the published literature from 2000 to 2022 describing the use of landiolol to treat atrial fibrillation in critical care settings, excluding both cardiac surgery and medical cardiac care settings. The primary outcome assessed was sinus conversion following landiolol treatment. **Results**: Our analysis identified 17 publications detailing the use of landiolol for the treatment of 324 critical care patients. While the quality of the data was generally low, primarily comprising non-comparative studies, landiolol consistently demonstrated similar efficacy in controlling heart rate and facilitating conversion to sinus rhythm in both non-surgical (75.7%) and surgical (70.1%) settings. The incidence of hypotension associated with landiolol use was 13%. **Conclusions**: The use of landiolol in critical care patients with new-onset atrial fibrillation exhibited comparable efficacy and tolerance in both non-surgical and surgical settings. Despite these promising results, further validation through randomized controlled trials is necessary.

## 1. Introduction

New-onset atrial fibrillation (NOAF) is a common complication in critically ill patients, with an incidence ranging from 4% to 46% [1,2,3]. Non-surgical patients generally have a lower risk, approximately 10%, except for those with septic shock, where the incidence can reach 30% [4]. In surgical patients, the standard rate of atrial fibrillation (AF) is around 10% [5]. However, higher rates, up to 40%, have been reported in cardiac surgery [5], lung surgery [6], and esophageal surgery [7]. The therapeutic goal in managing AF is to restore sinus rhythm in patients with hemodynamic instability [8]. For hemodynamically stable patients experiencing symptoms, the goal is to improve hemodynamics, typically through rate or rhythm control when the correction of trigger factors is insufficient [8]. Two studies have shown that outcomes, including cardiovascular events, are similar in patients treated with rhythm control or rate control [9,10]. However, since NOAF is often transient and spontaneously converts in most cases, reducing heart rate through rate control is a crucial step. Nevertheless, the control of heart rate in critically ill patients can lead to potential side effects that may counteract the benefits of medications [11]. Landiolol, an ultra-short-acting beta-blocker with a half-life of 4 min, a preferential negative chronotropic effect, and limited hypotensive impact, presents itself as an interesting agent for heart rate control in the critical care setting [12]. The objective of this review is to compare the use of landiolol and its hemodynamic profile in non-cardiac-surgery intensive care unit (ICU) settings and non-surgical ICU settings, with a specific focus on the rate of cardioversion. This analysis aims to contribute valuable insights into the potential benefits and risks associated with landiolol use in different critical care scenarios.

## 2. Materials and Methods

**Figure 1:**
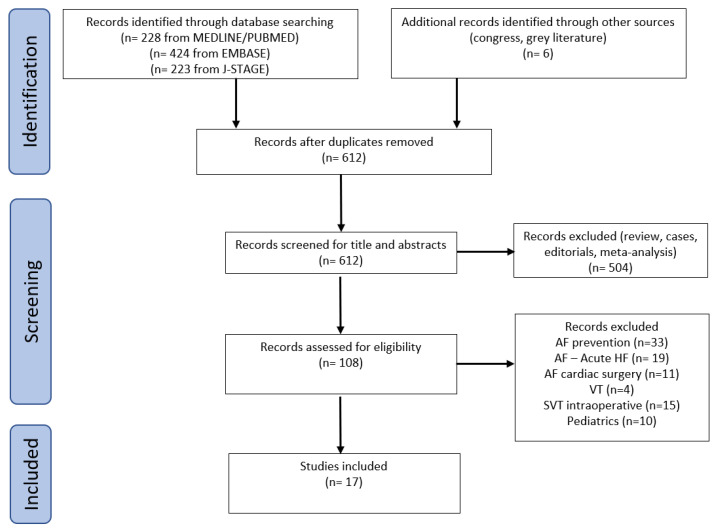
Flowchart of the study selection process.

We conducted a systematic review that conformed to the Preferred Reporting Items for Systematic Reviews and Meta-Analyses (PRISMA) standard (PROSPERO: CRD CRD42023410296) (Figure 1).

We conducted a search on the PUBMED, EMBASE, and J-STAGE databases using the keywords #landiolol OR #ono-1101 AND #atrial fibrillation OR #tachyarrhytmia OR #supraventricular tachycardia for the period ranging from the year 2000 to the year 2022, corresponding to the diffusion of landiolol around the world. We selected randomized controlled trials, cohort studies, and observational studies, while individual case reports were excluded. Only studies including adult patients hospitalized in an intensive care unit (ICU) were considered.

As landiolol was widely used in Japan, we also included the articles and abstracts published in Japanese. We excluded studies that included patients treated with landiolol for postoperative AF after cardiac surgery or patients with AF and acute heart failure managed with landiolol in cardiac care units.

The main outcome was the rate of cardioversion after the use of landiolol. Additional outcomes potentially available in studies, such as timing to sinus rhythm conversion, heart rate reduction rates, landiolol doses used, landiolol infusion duration, atrial fibrillation recurrences, and adverse events associated with landiolol, were also collected. The evaluation for inclusion was based upon whether studies were appropriate to address the relationship between the exposure (landiolol treatment) and the main outcome (conversion to sinus rhythm). Three reviewers performed the inclusion with guidance from the other researchers and based upon the eligibility criteria. Two review authors independently assessed the risk of bias of each study, using the evaluation grid to identify different categories of bias according to the ROBIN-E scale, to evaluate the confounding bias (pre-existing AF; antiarrhythmic pretreatment), measurement of exposure bias (medication dose or treatment duration), selection of participant bias (landiolol treatment allocation), post-exposure intervention bias (electric cardioversion or AA drug use), missing data bias (abstracts with a limited amount of detailed data; AF recurrence not reported), bias arising from measurement of the outcome (information on sinus rhythm conversion timing), and bias in the selection of the reported result (selective period analysis for SR conversion). Disagreements regarding the assessment of the risk of bias were adjudicated by a third author. There was no plan to conduct a meta-analysis as we anticipated the comparative data to be scarce and heterogeneous. A subgroup analysis was performed based upon the type of ICU (surgical, non-surgical).

The incidence of cardioversion is reported as the percentage of patients in sinus rhythm after conversion. An estimation of the mean rate of cardioversion was performed by calculating the sum of patients in sinus rhythm after landiolol treatment divided by the sum of patients in atrial fibrillation before landiolol treatment for each type of ICU. The lowest and highest conversion rates obtained for a given study are indicated in order to reflect the range. Calculation of the mean heart rate and reduction in blood pressure was not contemplated as we did not plan to contact authors to access patient data. However, hemodynamic findings are detailed for each study where available.

## 3. Results

Our database search yielded six publications where landiolol was used in a non-surgical setting to treat AF and ten publications where landiolol was used to treat postoperative AF in a non-cardiac surgery setting. One additional publication provided data on cardioversion for both surgical and non-surgical settings. Overall, ten surgical studies (two pulmonary surgical studies [13,14], six esophagectomy studies [15,16,17,18,19,20], two studies on other surgeries [21,22], and one unspecified surgical study [23]), six non-surgical studies (four sepsis studies [24,25,26,27], one septic shock study [28], and one SIRS study [29]), and one mixed ICU study [21] were included. The total number of patients treated with landiolol was 103 for the non-surgical setting and 221 for the surgical setting.

The studies included two randomized controlled trials (RCTs), three retrospective comparative studies, and twelve case series (a flowchart is shown in Figure 1). Furthermore, the first RCT included only 20% AF, the remaining being sinus tachycardia, and the second RCT was designed for the prevention of postoperative AF. Landiolol was used to control the heart rate of those patients that developed postoperative AF, and the results provide additional data on the conversion to sinus rhythm. Overall, the quality of the data was low and not sufficient for a meta-analysis. However, the risk of bias concerning the primary endpoint was judged to be “low” or of “some concern”, with no “high” risk of bias identified (Table 1).

**Figure 2:**
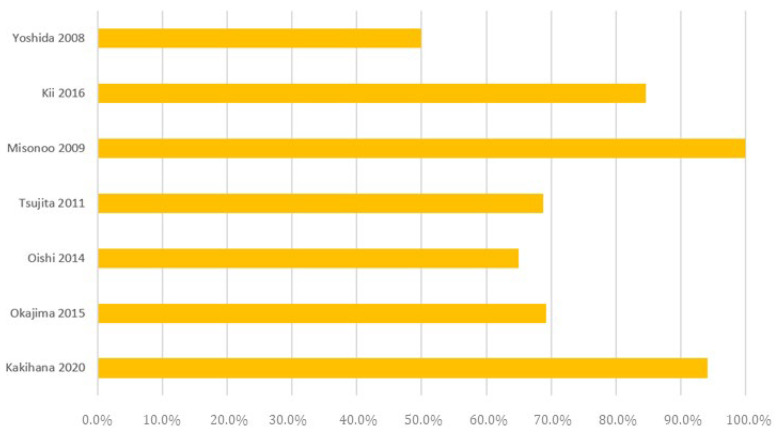
The rate of conversion to sinus rhythm in the medical ICU setting.

**Figure 3:**
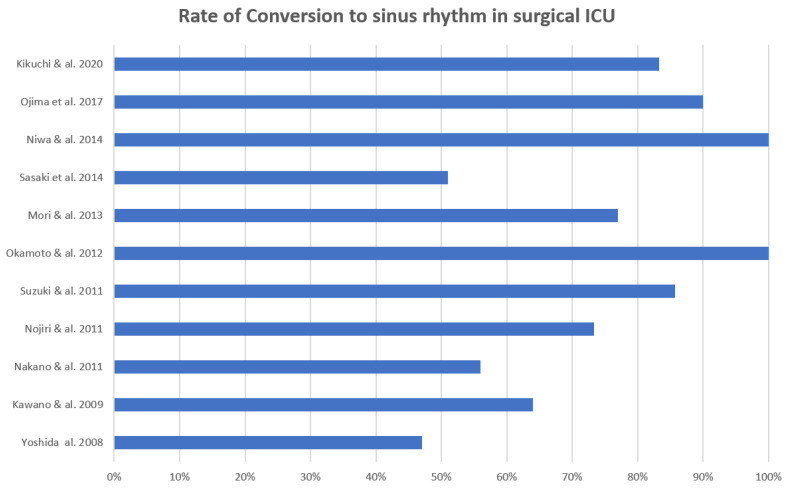
The rate of conversion to sinus rhythm in the surgical ICU setting (excluding cardiac surgery).

The rate of conversion of NOAF to sinus rhythm was similar in non-surgical and surgical patients, ranging from 50% to 100% (mean, 75.7%) and from 47% to 100% (mean, 70.1%), respectively (Figure 2 and Figure 3).

In most publications, the timing of the conversion to sinus rhythm was reported, with six studies [14,16,17,22,23,28] providing mean conversion times ranging from 1.8 h to 9.1 h. Additionally, five studies [13,18,24,25,26] presented conversion rates at 12 h or 24 h. Notably, detailed timing data revealed that the majority of patients converted within 12 h [13,14,16,18,24]. Landiolol treatment consistently resulted in a rapid and substantial reduction in heart rate with minimal impact on blood pressure. Twelve studies [13,14,15,16,17,18,19,21,22,24,26] provided data on the heart rate decrease, which ranged from −18% to −51% from baseline. Three additional studies [19,23,25,28] reported the percentage of patients achieving a heart rate target below 100 bpm (see Table 2). In studies with a control group, patients treated with landiolol exhibited an accelerated reduction in heart rate [14,16,24,27].

To control heart rate, landiolol dosages ranged between 5 and 10 µg/kg/min. There was a tendency to use higher doses in surgical settings (2 to 20 µg/kg/min) compared with non-surgical settings (0.4 to 12.5 µg/kg/min). Landiolol infusion typically lasted more than 24 h. Some studies reported a recurrence of AF after the landiolol infusion had ended, necessitating the resumption of or a transition to oral beta-blockers or alternative agents [13,15,16,17,18,28]. Higher dosages were associated with the prevention of AF recurrence in one study [23], and one study reported that 33% of patients continued on oral beta-blockers with no recurrence [21].

## 4. Tolerance and Adverse Events

The tolerance of landiolol was generally good, with the most frequent adverse events being hypotension and bradycardia, and no bronchospasms were reported. Only two case series [18,23] reported adverse events requiring landiolol discontinuation in two patients (one for hypotension [18] and two for hypotension and bradycardia [23]). This incidence (13%) aligns with the incidence (12%) of hypotension observed in the largest available randomized controlled trial (RCT) [27].

## 5. Discussion

The main results of the present systematic review are that landiolol consistently demonstrated similar efficacy in controlling heart rate and facilitating conversion to sinus rhythm in both non-surgical (75.7%) and surgical (70.1%) settings. The incidence of hypotension associated with landiolol use was 13%. The use of landiolol in critical care patients with new-onset atrial fibrillation exhibited comparable efficacy and tolerance in both non-surgical and surgical settings.

Sepsis, SIRS, and atrial fibrillation: AF is an early and prevalent complication during septic shock, affecting approximately 25–30% of admissions. The emergence of NOAF in septic shock patients is contingent upon the interplay of several factors, including the existence of an arrhythmogenic substrate, trigger factors, and modulating elements like the autonomic nervous system and inflammation [1]. Research has demonstrated triggered activity within the atrial musculature. An imbalance in the autonomic nervous system, specifically a shift toward sympathetic dominance and a reduction in heart rate variability, has been posited as a potential explanation for the onset of NOAF in individuals with sepsis [30]. This could lead to an elevated heart rate output, a phenomenon frequently observed in patients with sepsis. Unopposed and sustained tachycardia during this period is likely to further amplify calcium influx through L-type Ca^2+^ channels. This, in turn, results in significant shortening of the atrial refractory period and action potential duration, promoting triggered activity and facilitating the onset of atrial fibrillation. This mechanism appears to be heightened by beta-adrenergic stimulation post endotoxin application, influencing channel activity by extending the open time and abbreviating the close time of Ca^2+^ channels. These findings may elucidate the heightened sensitivity of cardiac pacemaker cells to the positive inotropic effects of adrenergic stimulation, potentially leading to the development of new AF episodes, particularly in the early stages of sepsis. Traditional cardiovascular risk factors typically do not elevate its occurrence, particularly in instances of NOAF. The inflammatory response during sepsis has been suggested to be a potential trigger. Landiolol has been associated with anti-inflammatory effects at low levels of high mobility group box 1, which is a key mediator of systemic inflammation [31]. A comprehensive retrospective analysis based on a population cohort, conducted by Walkey et al., demonstrated a considerable escalation in the risk of NOAF among patients with severe sepsis (n = 49,082) compared with those without severe sepsis. The odds ratio (OR) was 6.82, with a 95% confidence interval (CI) of 6.54–7.11 (*p* < 0.001) [32].

Several studies have indicated that the conventional risk factors associated with chronic atrial fibrillation in the general population may differ from those prevalent in septic patients experiencing NOAF. Factors contributing to the occurrence of NOAF in septic patients include conditions unrelated to chronic cardiovascular disease, such as an increased incidence of acute organ failure/dysfunction, mechanical ventilation, heightened comorbidities, and the utilization of pulmonary artery catheterization. Moreover, NOAF has been linked to additional factors, including a lower ejection fraction (EF), advanced age, elevated levels of troponin-HS and NT-pro-BNP, and a prolonged QRS duration. Using an updated definition of septic shock, Rabie et al. conducted a prospective study involving 100 septic shock patients, representing one of the largest series to date [33]. The patients underwent continuous monitoring using a three/five-lead monitor equipped with arrhythmia detection algorithms, alarms, and Holter recording capabilities throughout their ICU stay. The study revealed the development of NOAF in 29 patients (29%), with 22 patients (75.8%) experiencing a single occurrence and 7 patients (24.2%) encountering recurrent AF during their ICU stay [33]. This comprehensive monitoring approach, including Holter ECG, provides valuable insights into the occurrence and patterns of AF in the context of septic shock.

## 6. Postoperative Atrial Fibrillation in Non-Cardiac-Surgery Patients

Postoperative atrial fibrillation shares many similarities with NOAF occurring in non-surgical SIRS and sepsis patients. The three major common triggers are hyperadrenergic stimuli, oxydative stress, and inflammation, which can accumulate with pre-existing AF risk factors and other triggers [13]. The incidence of AF is directly related to the intensity and duration of these triggers, as reflected in the increasing incidence of NOAF in SIRS, sepsis, and septic shock, POAF in general, and thoracic and cardiac surgery. The very high incidence of AF observed in cardiac surgery stems from additional triggers, such as local inflammation and valvular disease, while lung surgery and gastrectomy are associated with right atrial stress or vessel dissection and the extent of thoracotomy or gastric dissection, which contribute to the high incidence of POAF [6,7,13]. Most POAFs are transient and will convert back to the sinus rhythm [8]. However, POAF’s impact on the prognosis is not negligible, with a prolonged ICU stay and an increase in morbidities such as rehospitalization or a higher risk for AF recurrence [2,8].

## 7. Management of NOAF/POAF in Critically Ill Patients

The adverse consequences of AF contribute to a deteriorating prognosis, even after adjusting for the severity of the underlying illness [30]. NOAF is linked to a higher mortality rate compared with pre-existing chronic AF. It is essential to distinguish upfront hemodynamically poorly tolerated AF, where urgent external electrical cardioversion (ECV) is imperative. However, in a recent study conducted in the ICU, the primary success rate of ECV was low (35%) and AF recurrence was frequent (reported in 38% and 62% of cases at 24 and 48 h, respectively) [34]. This underscores the importance of promptly initiating treatment after ECV to maintain the benefit of electrical reduction. Similarly, recurrence of the rhythm disorder may require the administration of an antiarrhythmic agent before electrical cardioversion in order to optimize its effectiveness. When the hemodynamics are not compromised by the rhythm disorder, the urgency is to wait! Indeed, a spontaneous reduction is not uncommon. A cardiology study comparing amiodarone to a placebo reported a 64% return to sinus rhythm at the 24th hour in the placebo group [35].

The guidelines for AF management are not always directly applicable to critically ill patients, as NOAF in individuals treated in an ICU differs in terms of rhythm disturbance causes compared with AF in the general community [36]. This distinction necessitates a tailored and context-specific approach to management. The question of medical treatment arises in the case of persistent or recurrent AF. The scarcity of studies conducted in the ICU does not allow us to favor one therapeutic approach over another. Therefore, one must turn to cardiology studies, where two main options emerge: rhythm control and heart rate control. Regardless of the chosen strategy, caution is warranted in the use of the most-recommended medications given the often unstable, polymedicated nature of patients and the challenging cardiac evaluation. There is no right or wrong choice, and the practitioner’s experience with a particular drug is crucial. Current guidelines recommend beta-blockers or calcium blockers as first-choice drugs to control heart rate in AF patients with LVEF > 40% (class I, level of evidence B) [36]. Amiodarone or beta-blockers, for rhythm or rate control, respectively, are reasonable choices, considering that both drugs also allow for rate control. Digoxin is an option to consider for rate control in cardiac dysfunction patients when beta-blockers or amiodarone are contra-indicated. In the presence of renal insufficiency, the intravenous administration of landiolol is an interesting alternative for rate control due to its short half-life and metabolism through esterase [12]. The consequences of a treatment useful in the acute phase but unnecessary in the long term can lead to side effects, especially with the use of certain medications (such as amiodarone). Amiodarone, commonly used in ICU settings, has potential toxicities and limited efficacy [37]. Recent meta-analyses have shown similar success rates between beta-blockers and amiodarone [38,39]. The rates of successful rhythm control using amiodarone varied from 30.0% to 95.2%, beta-blockers from 31.8% to 92.3%, calcium channel blockers from 30.0% to 87.1%, and magnesium from 55.2% to 77.8% [38]. The rate of successful rhythm control for digoxin was 55.6% in a single study [38]. A recent large cohort study comparing strategies to achieve rates below 110 bpm in AF septic patients showed that beta-blockers provided faster heart rate control at 1h, but there was no further difference at 6 h when compared with amiodarone, calcium channel blockers, and digoxin [40]. Hyperadrenergic stress, a common trigger in ICU patients, makes beta-blockers a relevant choice, and landiolol’s conversion rates are consistent with this mechanism. Comparatively to landiolol, esmolol is associated with hypotension [41,42], limiting titration, while landiolol has established efficacy and tolerance in critically ill patients [12]. When selecting a therapeutic option, clinicians must consider pre-existing treatments, especially beta-blockers. Discontinuation of beta-blockers can trigger NOAF [8], and reintroducing them for rate control is relevant. Future studies should consider patients’ beta-blockade status when stratifying or excluding specific groups.

## 8. Efficacy and Tolerance of Landiolol in Critically Ill Patients and Post-Surgery Patients

Few studies have directly compared landiolol with other agents for treating NOAF in the critical care setting. Existing research is limited to one RCT in septic patients [28] that showed no difference for AF conversion between landiolol and the control in the subgroup of 29 AF patients. There are no RCTs comparing landiolol with another agent for treating postoperative AF in non-cardiac-surgery patients. The four retrospective studies, which included a historical group control principally using a calcium blocker or digoxin, tend to show a faster conversion and higher rate of conversion to SR for the landiolol group [14,16,24,29]. In contrast, many RCTs have assessed landiolol for postoperative AF prevention in ICU patients [12,43,44,45,46]. Although recommendations for managing AF are mainly derived from acute cardiac care units or cardiac surgery, critically ill patients have specific risk factors that need consideration. In this current systematic review, we observed that the use of landiolol was linked to significant efficacy and a low incidence of side effects. Importantly, due to the very short half-life of landiolol, any occurrence of hypotension or a decrease in cardiac output can be promptly reversed by discontinuing the drug. Likewise, the administered doses were low and similar to the dosage range of 1 to 10 µg/kg/min recommended in cardiac dysfunction patients [47,48,49]. Our results confirm the results of the studies using landiolol to treat NOAF in the postoperative setting of cardiac surgery [50,51,52] and those of the J-Land3S study [28,53]. It is to be noticed that patients included in the J-Land3S study had a preserved EF, which was maintained throughout the period of study. In the recent Stress L study, there was no cardiac output monitoring or echography measurement to monitor heart rate control, which did not allow us to distinguish patients benefitting from rate control from those potentially harmed by excessive beta-blockade [54]. Hence, landiolol should be used while monitoring the cardiac output and titrating for a decrease in heart rate. The results of this study show that conversion to sinus rhythm is obtained in two-thirds of patients, while a transition to oral beta-blockers may prevent AF recurrence.

## 9. Limitations

The main limitation of the present review is the lack of high-quality studies, with no RCTs comparing landiolol with other therapeutic options in non-cardiac surgery or medical ICU settings. Indirect comparative studies have diverse comparators, making it challenging to determine the best strategy. However, the dosing scheme for landiolol indicates consistent heart rate control in critically ill patients.

Most case series focused on esophagectomy and lung surgery, with limited representation of general surgeries. Non-surgical ICU settings accounted for one-third of patients, while surgical settings comprised two-thirds. Despite these limitations, conversion rates to sinus rhythm were consistent across settings, suggesting that landiolol accelerates the natural conversion of new-onset atrial fibrillation.

## 10. Conclusions

After two decades of use, predominantly in Japan, a limited number of studies have focused on landiolol for treating AF in critically ill patients. Existing data show consistent dose–response patterns and support the good tolerance of landiolol. However, more controlled studies in non-cardiac surgery or medical ICU settings are needed to position landiolol against other available treatments for managing critically ill patients with atrial fibrillation.

## Figures and Tables

**Table 1:** Risk of bias for the primary endpoint using the ROBIN-E scale.

Study Reference	Confounding Bias	Measurement of Exposure Bias	Selection of Participant Bias	Post-Exposure Intervention Bias	Missing Data Bias	Outcome Measurement Bias	Bias in the Selection of the Reported Result
Yoshida 2008	Low	Low	Some concern	Some concern	Some concern	Some concern	Low
Kawano 2009	Low	Low	Some concern	Low	Low	Some concern	Some concern
Nakano 2011	Some concern	Low	Low	Some concern	Low	Low	Some concern
Nojiri 2011	Low	Low	Low	Low	Low	Low	Low
Suzuki 2011	Some concern	Low	Some concern	Low	Low	Low	Low
Okamoto 2013	Some concern	Low	Low	Some concern	Some concern	Low	Some concern
Mori 2013	Low	Low	Low	Low	Low	Low	Low
Niwa 2014	Low	Low	Low	Low	Low	Low	Low
Ojima 2017	Low	Low	Low	Low	Some concern	Low	Low
Kikuchi 2020	Low	Low	Low	Low	Some concern	Some concern	Low
Misonoo 2009	Low	Some concern	Some concern	Low	Low	Some concern	Some concern
Tsujita 2011	Low	Low	Low	Low	Low	Low	Some concern
Sasaki 2014	Some concern	Low	Low	Low	Low	Low	Some concern
Oishi 2014	Some concern	Low	Some concern	Low	Low	Low	Some concern
Kii 2016	Low	Low	Low	Low	Low	Some concern	Low
Okajima 2015	Low	Low	Low	Low	Low	Low	Low
Kakihana 2020	Some concern	Low	Low	Some concern	Low	Low	Some concern

**Table 2:** List of included studies.

Reference	Study Design/Objective	Participants	Protocol for Landiolol Use	Main Results		Other Information
				AF Conversion	Hemodynamic	
Yoshida et al. 2008 [21]	Retrospective single-center study Investigate the clinical use and efficacy of LDL in an intensive and coronary care unit (indication, infusion rate, HR, BP, catecholamine use, and oral BB transition).	LDL was administered to 80 patients, including 27 AF patients in a surgical setting (n = 17) and a non-surgical setting (n = 10)	LDL initial infusion rate: 5–10 μg/kg/min titrated in increments of 1–2 μg/kg/min Median dose: 5 μg/kg/min Median duration: 2 days	47% (8/17) SR conversion (post-surgery) and 50% (5/10) SR conversion (medical ICU)	35% and 20% of the HR target (−21% bpm) for the surgical and medical ICU setting, respectively	−17.4% HR decrease in patients on catecholamines and a −26% HR decrease in patients without catecholamine support (NS)
Kawano et al. 2009 [23]	Retrospective single-center study Investigate the effects of LDL on rhythm control and rate control for POAF.	n = 36 AF n = 25 Paroxysmal AF (PAF) and n = 11 Chronic AF (CAF)	LDL: 8.5 ± 7.9 μg/kg/min Prevention of recurrence of AF tends to be associated with a higher landiolol dosage during the maintenance phase (5.5 +/− 4.1 mcg/kg/min vs. 2.8 +/− 0.7 mcg/kg/min, *p* = 0.09) Duration: NA	64% (16/25) SR conversion within 3 h in PAF patients.	HR decrease (<100 bpm) in 64% of PAF patients and 82% of CAF patients. Landiolol infusion discontinued due to unexpected bradycardia and hypotension in 2 of 36 patients (5%)	Factors associated with SR restoration: - a higher initial landiolol dose (10.0 +/−9.0 mcg/kg/min vs. 5.6 +/− 4.0 mcg/kg/min, *p* < 0.05); - a lower frequency of coexisting heart failure (35% vs. 88%, *p* < 0.05); - administration of catecholamines (29% vs. 88%, *p* < 0.05).
Nakano et al. 2011 [13]	Two-center prospective observational cohort study Evaluate the efficacy of LDL for POAF	n = 25 patients undergoing pulmonary resection	LDL: 60 μg/kg/1 min + 5–10 μg/kg/min Patients (duration): 5 (24–72 h), 4 (3–7 days), and 4 (>7 days)	48% (14/25) SR conversion within 24 h	−37% HR decrease −8.5% BP decrease	No side effects, including those related to the circulatory and respiratory systems
Nojiri et al. 2011 [14]	Retrospective single-center study Evaluate the safety and efficacy of low-dose LDL for POAF in lung cancer surgery (SBP, DBP, HR, and oxygen saturation at baseline and 30 min, 2 h, and 12 h after starting medication). Time to restoration of SR	n = 15 (LDL) n = 15 (CTL) Exclusion criteria: history of AF, antiarrhythmic drug use including β-blockers, thyroid dysfunction, renal failure requiring hemodialysis, repeated pulmonary resection, and recent (<1 month) angina pectoris or myocardial infarction.	LDL: 5–10 μg/kg/min Infusion duration: 8.1 ± 11.0 h followed by 2.5–5.0 mg of carvedilol orally each day for 1 month CTL: 0.25 mg of digoxin and 5 mg of verapamil I.V. loaded every 12 h for 1 day and then 0.125–0.25 mg of digoxin and 120 mg of verapamil orally each day for 1 month	SR conversion of 73% (11/15) within 24 h (53% at 2 h) for LDL 53% (8/15) SR conversion (20% at 2 h) for CTL	−40% HR decrease No impact on SBP/DBP. HR was significantly lower in LDL vs. CTL at 30 min, 2 h, and 12 h. (*p* < 0.05)	Also included a subgroup with the following landiolol regimen: loading at 20–60 μg/kg/min for 20 min + a decrease at 1–5 μg/kg/min No postoperative deaths, thromboembolic events, or congestive heart failure events associated with AF in either group. One (7%) case of pneumonia in the LDL group. Four (27%) cases of pneumonia, two (13%) cases of hypotension, and one (7%) case of acute respiratory distress syndrome in the CTL group. All patients recovered with treatment.
Suzuki et al. 2011 [18]	Retrospective case series Investigate LDL’s effects on hemodynamics and the antiarrhythmic effects other than BB failure in esophageal cancer surgery patients with POAF after treatment with antiarrhythmic drugs	n = 7 esophageal cancer surgery patients Initial AF management before LDL: fluid load, sedation, analgesia, and treatment with antiarrhythmic drugs	LDL: 10.8 mcg/kg/min Infusion duration: NA Prior AA drugs in five cases, digoxin in five cases, disopyramide phosphate in two cases, cibenzoline in one case, adenosine triphosphate in one case, and magnesium sulfate in two cases.	86% (6/7) SR conversion within 24 h (29% at 1 h, 57% at 6 h)	−51% HR decrease (63% achieved the HR target of 100 bpm)	No bronchospasm In 3/7 cases, a recurrence of PAF was observed after the administration of landiolol ended
Okamoto et al. 2013 [17]	Retrospective single-center study Describe the effects of LDL on tachyarrhythmia in postoperative esophagectomy (SBP, DBP, HR, and oxygen saturation at baseline and 1 h, 2 h, 4 h, 24 h, and 48 h after starting medication)	n = 38 patients with AF after endoscopic esophageal cancer surgery 28 patients treated with LDL 10 patients treated with digoxin or a calcium antagonist	LDL: started at 3 to 5 mcg/kg/min Mean LDL dose: 4.1 ± 2.4 mcg/kg/min Infusion duration: 110.8 ± 71.2 h	100% (28/28) SR conversion Time to conversion: 9.1 h ± 14.0 Time to conversion in the dixogin/calcium antagonist: 22.2 h ± 20.3	−18% HR decrease −3% SBP decrease −2% DBP decrease	No asthma crisis occurred after administration in four patients with bronchial asthma. Three cases of AF recurrence after LDL discontinuation. No significant difference in postoperative complications between the LDL group and the non-tachycardic historical control
Mori et al. 2013 [15]	Single-center prospective observational cohort study Evaluate the efficacy and safety of LDL for tachyarrhythmia in postoperative esophagectomy	n = 13/74 (18%) esophageal cancer patients that developed AF after transthoracic esophagectomy Exclusion criteria: history of heart disease with NYHA ≥3, postoperative use of another BB or antidepressant, and marked liver or kidney dysfunction	LDL: loading for 1 min at a dose of 60 mcg/kg/min + 10 mcg/kg/min up to 40 mcg/kg/min Mean LDL dose: 26.9 ± 12.5 mcg/kg/min Infusion duration: at least until the HR target was reached (−20%) or SR conversion	76.9% (10/13) SR conversion in less than 1 h 82% (9/11) in AF and 50% (1/2) in PSVT	−38% HR decrease (77% on the HR target of <100 bpm) −10% MAP decrease	2 MAP < 80 mmHg and 2 MAP with a 30% decrease = > hypotension not necessitating a vasopressor or discontinuation No bronchospasm or ischemia 6/11 AF relapse (55%) necessitating BB resumption or an alternative
Niwa et al. 2014 [16]	Single-center retrospective cohort study Evaluate the efficacy and safety of LDL for tachyarrhythmia in postoperative esophagectomy	n = 32/231 (10.8%) esophageal cancer patients that developed AF after transthoracic esophagectomy Exclusion criteria: eight patients were excluded (five receiving LDL and digoxin, CCB, or disopyramide and three who were not treated)	n = 11 (LDL): the mean dose started at 6.5 ± 3.4 then increased to 7.7 ± 4.4 mcg/kg/min Infusion duration: 38 ± 42 h Eight patients with NOAF, one with chronic AF, and two with sinus tachycardia n = 13 (CTL): alone or in combination with digoxin (n = 11), verapamil (n = 6), or disopyramide (n = 3)	LDL: 62.5% (5/8) at 2 h and 100% (8/8) at 12 h Mean SR conversion time: 3.6 h ± 6.6 CTL: 7.7% (1/13) at 2 h and 46% (6/13) at 12 h Mean SR conversion time: 23.3 h ± 5.2 SR conversion was faster at 2 and 12 h (*p* < 0.05)	The HR reduction % at 1 h was higher in LDL compared with CTL: −28.5 ± 4.4% vs. 12.3 ± 3.5% (*p* = 0.011) The SBP and DBP reduction % was similar in the LDL and CTL groups: SBP: −14.3 ± 8.3% vs. −13.5 ± 14.5% (*p* = 0.883) DBP: −16.6 ± 7.1% vs. −9.5 ± 9.8% (*p* = 0.061)	AF recurred in one patient in the LDL group and three patients in the CTL group; one LDL patient experienced an episode of bradycardia/hypotension. No bronchospasm or ischemia in either group.
Kikuchi et al. 2020 [19]	Single-center retrospective cohort study Evaluate the effectiveness of LDL for treating tachyarrhythmia after esophageal cancer surgery (SBP, DBP, HR, and oxygen saturation at baseline and each hour after starting medication) Identify AF risk factors	n = 19/141 (13.5%) esophageal cancer patients that developed AF after thoracotomy or thoracoscopic esophagectomy Patients without tachyarrythmia (n = 122) were used as the CTL for identifying AF risk	LDL: 60 μg/kg/1 min + 20 μg/kg/min Infusion duration: NA	83.3% (10/12) SR conversion Timing: NA Length of hospital stay not significantly longer in patients with postoperative tachyarrhythmia (*p* = 0.0056).	75% reached the HR target of <100 bpm No impact on SBP	No deterioration of respiratory conditions, such as bronchial stenosis, was observed Risk factors for tachyarrhythmia: preoperative ECG abnormalities (*p* = 0.0001); history of CV disease (*p* = 0.0061); history of oral CV medicine (*p* = 0.0007); long-term surgery (*p* = 0.01). Presence or absence of preoperative chemotherapy (*p* = 0.59) and history of cerebrovascular disease (*p* = 0.134) were not significant factors.
Ojima et al. 2017 [20]	Single-center randomized, double-blind, and placebo-controlled trial Determine whether LDL is effective and safe for the prevention of AF after oesophagectomy	100 patients scheduled for transthoracic oesophagectomy receiving landiolol (n = 50) or a placebo (n = 50) for AF prevention 20 patients (5 LDL, 15 CTL) that developed AF	Patients that developed POAF all received LDL: 3 to 5 μg/kg/min Infusion duration: NA	90% (18/20) SR conversion Median duration for POAF: 27.5 h [1–180 h]	NA for landiolol use as a POAF treatment. When used for POAF prevention, LDL effectively suppresed postoperative HR, but the decrease in BP was not harmful.	18 of 20 patients returned to SR; no electrical cardioversion needed. LDL at 3 μg/kg/min for 72 h reduced the incidence of POAF (5/50) vs. the placebo (15/50), *p* = 0.012 The overall incidence of postoperative complications was significantly lower in the LDL group (*p* = 0·046).
Kakihana et al. 2020 [28]	Multi-center randomized, open-label, and controlled trial Investigate the efficacy and safety of LDL for treating sepsis-related tachyarrhythmias Primary outcome: proportion of patients with an HR of 60–94 bpm at 24 h after randomization SBP, DBP, and HR at 24 h, 48 h, 72 h, and 96 h after initiation of treatment. Tachyarrhythmia and safety outcomes at 168 h after randomization	Baseline SR patients n = 57 (LDL) n = 63 (CTL) Baseline AF patients n = 17 (LDL) n = 12 (CTL)	LDL: 5.3 ± 5.2 μg/kg/min Infusion duration: 58.2 h ± 50.4 h Additional AA drugs LDL: group I-AA (n = 4), BB (n = 1), amiodarone (n = 3), CCB (n = 2), digoxin (n = 2) CTL: group I-AA (n = 5), BB (n = 11), amiodarone (n = 7), digoxin (n = 1)	SR conversion: 94.1% (16/17) at 168 h SR conversion in the control group: 83.3% (10/12)	41.2% (7/17) of LDL patients reached an HR of <95 bpm at 24 h vs. 41.4% (5/12) in the CTL group 47.1% of the LDL group developed an adverse event vs. 50% of the CTL group.	In the SR baseline group, 10.5% (6/57) of LDL patients and 27% (17/63) of CTL patients developed NOAF at 168 h Overall, a lower incidence of NOAF at 168 h after randomization in the LDL vs. CTL groups (9% (7 of 75) vs. 25% (19 of 75)), *p* = 0·015 Adverse events led to LDL discontinuation in nine patients (12%). Hypotension was the most frequent adverse event, which either resolved or improved even in serious cases after taking appropriate measures, such as a dose reduction, LDL withdrawal, or the administration of catecholamine.
Okajima et al. 2015 [24]	Historical-cohort, single-center, interventional, and inter-subjective comparison study Investigate the safety and efficacy of LDL in controlling the HR of SVTs in severe sepsis patients SBP, DBP, and HR at 1 h, 8 h, and 24 h after initiation of tachyarrhythmia. Heart rhythm and conversion to sinus rhythm. Pulmonary arterial pressure, central venous pressure (CVP), cardiac output, and cardiac index (CI) were measured if a pulmonary arterial catheter was inserted. Systemic vascular resistance index (SVRI)	n = 61/163 (37.4%) septic patients with tachyarrhythmia, n = 39 (LDL group) and n = 22 (CTL group) Intra-abdominal infection was higher (*p* < 0.05) and urinary tract infection was lower (*p* < 0.05) in the LDL group compared with the CTL group	LDL: 5.5 ± 4.1 mcg/kg/min Infusion duration: 80.7 h ± 78.5 h CTL: calcium channel blockers and antiarrhythmic agents	LDL: 69.7% (27/39) SR conversion within 24 h (25.6% at 1 h, 55.3% at 8 h) CTL: 36.4% (8/22) SR conversion within 24 h (0% at 1 h, 18.2% at 8 h) SR conversion was observed more frequently in the LDL group than in the CTL group at each point (Figure 1, *p* < 0.01 at 8 h; *p* < 0.05 at 24 h).	HR drop: −18% (1 h); −38% (24 h) HR reduction: 145 ± 14 to 90 ± 20 bpm at 24 h No impact on MAP At 24 h after the initiation of tachyarrhythmia, landiolol reduced the HR dramatically (from 145 ± 14 bpm to 90 ± 20 bpm, Figure 1). There was a lower degree of HR reduction in the CTL group (from 136 ± 21 bpm to 109 ± 18 bpm) compared with the LDL group	Greater HR decrease vs. the control group. Baseline diastolic pulmonary arterial pressures were similar between groups and did not change. In the LDL group, the baseline CI was lower and did not decrease compared with the control group.
Tsujita et al. 2011 [29]	Retrospective case series Evaluate the effectiveness of landiolol in SIRS patients with tachyarrhythmia Part 1: SBP, DBP, HR, CVP, SVI, and SVRI at baseline and 2 h, 4 h, and 6 h after starting medication Part 2: delta SBP, DBP, HR, SR conversion rate, and timing when comparing LDL to other agents	167 patients treated with LDL, digoxin, cibenzoline, and verapamil for arrhythmia, among which n = 16/37 (LDL), n = 23/98 (digoxin), n = 19/56 (cibezoline) and n = 21/47 (verapamil) met the SIRS criteria for inclusion.	LDL: 0.5 to 5 mcg/kg/min Infusion duration: 139 h ± 118 h CTL: Digoxin: 0.125–0.250 mg I.V. Cibenzoline: 35–75 mg Verapamil: 2.5–5.0 mg	Part 1: 68.8% (11/16) SR conversion within 1.8 h ± 1.6 91% (10/11) AF recurrence Part 2: SR conversion LDL: 60% (10/15) Digoxin: 26% (6/23) Cibenzoline: 63% (12/19) Verapamil: 19% (4/21) Time to conversion for digoxin: 250 ± 91 min. Less than 90 min for cibenzoline and verapamil	81% of patients reached the HR target −41% HR −6% SBP −9% DBP LDL had a significantly lower heart rate effect compared with digoxin, cibenzoline, and verapamil (*p* < 0.05) SBP and DBP were both mildly reduced, and there was no significant difference compared with the other agents	In 2/16 cases, infusion was discontinued due to an AE (hypotension) In both cases, the BP returned to the original BP within 30 and 80 min of infusion discontinuation, respectively. Treatment of AF recurrence: landiolol resumption (n = 3), carvedilol (n = 4), bisoprolol (n = 2), verapamil (n = 1)
Misonoo et al. 2009 [26]	Retrospective case series Evaluate the effectiveness and safety of landiolol in septic patients with tachyarrhythmia MAP, HR, CVP, and ECG blood gas at baseline and 12 h Adverse events	21 septic patients, among which AF patients (n = 8) and VT patients (n = 2) received an LDL infusion for at least 24 h	LDL: 3.7 ± 2.5 mcg/kg/min Infusion duration: 48 h	100% (8/8) SR conversion at 12 h	76% of patients reached the HR target (<95 bpm) at 12 h −30% HR 121 ± 20 to 85 ± 14 bpm The MAP, CVP, SpO2, and PaO2–FiO2 ratio did not change significantly	Low SBP (<90 mmHg) was observed in some patients. No bradycardia Two VT patients also converted.
Kii et al. 2016 [27]	Retrospective study Evaluate the safety and efficacy of LDL for patients with septic shock MAP, HR, ECG, lactate, and fluid	19 septic patients, among which were AF patients (n = 13) and sinus tachycardic patients (n = 6)	LDL: 2.6 ± 1.9 μg/kg/min Infusion duration: 5.6 ± 3.9 days	84.6% (11/13) SR conversion	HR decreased significantly (*p* < 0.0001). No significant change in BP before and after administration (*p* = 0.1045)	Eight cases in which noradrenaline was used concomitantly, and the dose was 0.12 ± 0.07 μg/kg/min The 6 h fluid infusion volume was 39.3 ± 30.3 mL/kg. The 24 h fluid infusion volume was 123.5 ± 79.1 mL/kg. The 24 h lacate clearance was 21.9 ± 40.6%.
Sasaki et al. 2014 [22]	Retrospective study Medical and surgical intensive care setting Evaluate the effects of LDL on arrhythmia MAP, HR, and SR conversion rate	95 ICU patients with arrhythmia, among which were PAF patients (n = 51), PSVT patients (n = 16), persistent AF patients (n = 15), and Aflut patients (n = 2)	LDL: 4.3 ± 2.9 μg/kg/min Infusion duration: 41.4 h ± 50.1 h LDL was used as a first-line treatment in 72% of cases and a second-line treatment in 28% of cases after verapamil (n = 12), digoxin (n = 8), disopyramide (n = 7), cibenzoline (n = 3), pilsicainide (n = 1), and amiodarone (n = 1)	51% (26/51) SR conversion Conversion time: 3.8 h ± 6.7	−30% HR decrease No impact on MAP Regardless of whether a vasopressor agent was used prior to administration, a significant decrease in BP was not seen at the start of administration and 1 and 6 h after dosing. The HR significantly decreased 1 h after LDL administration and lasted for 6 h after dosing	A mixed ICU including 15% non-surgical patients, 19% gastrointestinal surgery patients, 30% large-vessel surgery patients, and 26% heart surgery patients.
Oishi et al. 2014 [25]	Retrospective study on tachyarrhythmias in critically ill patients with sepsis Compare patients that developed de novo tachyarrhythmias to patients without tachyarrhythmias during ICU admission Compare the incidence of arrhythmias in septic patients as well as the response to treatment.	43% (63/147) of patients developed de novo arrhythmias: AF, 60; Aflut; PSVT, 7; VT, 3 Exclusion criteria: ICU stay < 24 h Hemofiltration Trauma History of AF	Digoxin (n = 55): 0.125 to 0.250 mg LDL added to digoxin (n = 24): 0.4 to 12.5 μg/kg/min Milrinone use and norepinephrine use were significantly higher in the arrhythmia group	In the 60 patients with AF: 65% (39/60) SR conversion within 24 h (50% at 6 h, 57% at 18 h) PSVT and VT: SR conversion for 3 patients at <6 h	78% reached the HR target (24 h) HR control was achieved in 58% (35/60) of patients at 6 h and 66% (40/60) of patients at 18 h	Landiolol was used in 24 patients in association with digoxin. Patients not converting to SR were associated with higher mortality. Significantly higher ICU mortality (22%; 14/63 cases) and in-hospital mortality (35%; 22/63 cases) in the arrhythmic group compared with the non-arrhythmic group (10%; 8/84 cases and 19%; 16/84 cases, respectively).

NA, not available; AF, atrial fibrillation; AFlut, atrial flutter; PAF, paroxysmal atrial fibrillation; POAF, postoperative atrial fibrillation; LDL, landiolol; CTL, control; HR, heart rate; SBP, systolic blood pressure; MAP, mean arterial pressure; SVT, supraventricular tachyarrhythmia; PSVT, paroxysmal supraventricular tachyarrhythmia; BB, β-blocker; AA, antiarrhythmic; CCB, calcium channel blocker; I.V., intravenously; CV, cardiovascular; PAP, pulmonary arterial pressure; CVP, central venous pressure; CO, cardiac output; CI, cardiac index.

## Data Availability

The datasets analyzed during the current study are available from the corresponding author on reasonable request.
